# Applying population‐specific incidence prevalence ratio benchmarks to monitor the Australian HIV epidemic: an epidemiological analysis

**DOI:** 10.1002/jia2.26399

**Published:** 2024-12-04

**Authors:** Jonathan M. King, Hamish McManus, Richard T. Gray, Steven J. Nigro, Jana Sisnowski, Timothy Dobbins, Benjamin R. Bavinton, Andrew E. Grulich, Kathy Petoumenos, Jane Costello, Skye McGregor

**Affiliations:** ^1^ The Kirby Institute University of New South Wales Sydney New South Wales Australia; ^2^ Health Protection NSW NSW Health Sydney New South Wales Australia; ^3^ Communicable Disease Control Branch SA Health Adelaide South Australia Australia; ^4^ School of Population Health University of New South Wales Sydney New South Wales Australia; ^5^ Positive Life NSW Sydney New South Wales Australia

**Keywords:** HIV epidemiology, incidence, key and vulnerable populations, men who have sex with men, people who inject drugs, prevalence

## Abstract

**Introduction:**

Due to a lack of robust population denominators, Australia is unable to accurately monitor changes in the HIV epidemic for some populations. The ratio of HIV transmission relative to the number of people with HIV (an incidence prevalence ratio, or IPR) can measure such changes. The IPR is measured against an IPR benchmark derived from post‐HIV acquisition life expectancy, to indicate whether an HIV epidemic is shrinking or growing. Using IPRs and Australia‐specific IPR benchmarks, this study aims to describe the Australian HIV epidemic among three groups: men with HIV attributed to male‐to‐male sex, women with HIV and people with HIV attributed to injection drug use.

**Methods:**

Using mathematical modelling derived from HIV notifications, cohort and administrative data, IPRs were generated for each of the three groups. These IPRs were compared with IPR benchmarks derived from post‐HIV acquisition mortality estimates using abridged life tables for men, women and people who inject drugs. The IPR benchmark for men was applied to people with HIV attributed to male‐to‐male sex. Trends in the IPR over time were described for each reported population from 2015 to 2022.

**Results:**

Overall, the IPR fell by 80%, from 0.040 (range: 0.034−0.045) in 2015 to 0.008 (range: 0.003−0.013) in 2022 and fell below the benchmark (0.022) in 2020. Among people with HIV attributed to male‐to‐male sex, the IPR fell by 85%, from 0.041 (range: 0.034−0.047) in 2015 to 0.006 (range: 0.003−0.024) in 2022 and fell below the benchmark (0.022) in 2020. Among women with HIV, the IPR fell by 56%, from 0.032 (range: 0.026−0.039) in 2015 to 0.014 (range: 0.003−0.029) in 2022 and fell below the benchmark (0.022) in 2019. Among people with HIV attributed to injection drug use, the IPR fell by 61%, from 0.036 (range: 0.022−0.047) in 2015 to 0.014 (range: 0.002−0.057) in 2022 and fell below the benchmark (0.028) in 2019.

**Conclusions:**

Australian IPRs in all populations examined have dropped below the level required to sustain the HIV epidemic at current levels. By applying this method in other contexts, the changing scale of HIV epidemics may be better described for populations lacking robust population denominators.

## 1 INTRODUCTION

The UNAIDS 95‐95‐95 Fast‐Track targets aim to have 95% of people living with HIV being aware of their HIV status, 95% of those diagnosed with HIV receiving antiretroviral treatment (ART) and 95% of those on treatment having a suppressed HIV viral load [1]. Modelling shows that achieving all three targets by 2030 will reduce HIV incidence by 90% from 2010 levels, a monumental achievement in the effort to end the HIV epidemic. However, in the absence of robust population denominators, changes in HIV transmission relative to population size are difficult to measure. Therefore, other metrics are needed to track levels of HIV transmission and changes in the scale of the HIV epidemic. These metrics can help evaluate the impact of public health measures and inform service provision. One measure, the incidence prevalence ratio (IPR), has been used by UNAIDS and others to track changes in HIV epidemics across countries and regions [2, 3, 4, 5, 6, 7, 8].

The IPR is defined as the number of HIV transmission events divided by the estimated number of people in that population living with HIV [9]. In other words, the IPR in a given population is the number of HIV transmissions divided by the number of people living with HIV. An IPR equilibrium benchmark can be used to compare the IPR with the rate of replacement for the population living with HIV [9]. Until a cure for HIV is developed, the length of time a person lives with HIV equates to the remaining lifespan of the person following HIV acquisition. For the HIV epidemic to decline, the average number of transmission events per person with HIV should be fewer than one. Therefore, when the epidemic is at equilibrium, the benchmark value is equal to 1/D, where 1 equates to one HIV transmission event and D is the lifespan of the individual post‐acquisition [9]. UNAIDS estimates that globally, D equates to 33 years meaning that the global IPR benchmark is 0.03 [10].

Although this benchmark may be appropriate when looking at global trends, it fails to account for wide variations in life expectancy across regions as well as the average age at HIV acquisition [11]. For example, according to the World Bank, in 2021, the average life expectancy in high‐income countries was 80 years, whereas in low‐income countries, the life expectancy was 62 years [12]. With an increasing number of countries achieving high rates of treatment coverage (>95%), the life expectancies of people with HIV in these countries are nearing that of the general population [13, 14]. Therefore, people with HIV in countries with high overall life expectancy and high treatment coverage are likely to have considerably higher life expectancies than the global average of people with HIV.

Australia has universal healthcare that allows for highly subsidized ART for all people with HIV residing in Australia, regardless of visa status [15]. This setting contributes towards the high proportion of people with HIV on ART and hence, the life expectancy for people with HIV in Australia is likely higher than the global average [16, 17]. Therefore, an Australia‐specific IPR benchmark would likely be more appropriate to apply to Australian IPR estimates and be valuable in measuring changes in the scale of Australia's HIV epidemic. By describing trends in the IPR for populations, including population‐specific IPR benchmarks where appropriate, gaps in Australia's response to the HIV epidemic may be revealed.

For populations within regions, it may not be appropriate to use the same benchmark as the region overall [8]. For example, among people living with HIV and a history of injection drug use, increased mortality associated with HIV/AIDS has been observed, likely attributable to limitations in access and adherence to ART [18]. Also, greater non‐HIV/AIDS‐attributed mortality has been observed among people with a history of injection drug use compared with people without a history of injection drug use, attributable to higher levels of homelessness, poor mental health and lower levels of healthcare engagement [19].

To the best of our knowledge, this is one of the first studies to describe the HIV IPR measured against a country‐specific IPR benchmark. Also, this is one of the first studies to describe population‐specific IPRs with population‐specific IPR benchmarks.

## 2 METHODS

Incidence and prevalence estimates used to calculate the IPR estimates were generated using the methods for producing Australia's HIV diagnosis and care cascade. Estimates were generated for the years 2015−2022, inclusive. These methods have been detailed extensively in previous publications [17, 20–22]. In summary, the annual estimate for the number of people living with diagnosed HIV was obtained by summing annual notifications and adjusting for duplicates, mortality, immigration and emigration using available registry, cohort and administrative data. We then applied the European Centre for Disease Prevention and Control (ECDC) HIV Modelling Tool (version 1.3.0) to estimate the annual number of HIV acquisitions (HIV incidence) with the percentage undiagnosed used to inflate the diagnosed estimate to the overall number living with HIV (HIV prevalence) [23]. IPR estimates were generated for all people living with HIV, women living with HIV, men living with HIV attributed to male‐to‐male sex and people living with HIV attributed to injection drug use.

Using HIV notification records with a diagnosis date of between 1 January 2015 and 31 December 2020, we generated Australian IPR benchmarks using mortality estimates from Australia's National HIV Registry linked with the Australian National Death Index. Mortality estimates in 5‐year age bands with an open age band at ≥85 years were evaluated using Royston‐Palmer parametric survival models adjusted for sex and injecting drug use [24]. Expected remaining years of life following HIV diagnosis was derived from abridged life tables based on estimated mortality closed out at 80 years which were generated using a method adapted from a previously validated approach described elsewhere [13, 14]. To reflect the introduction of Test and Treat guidelines, which recommends prompt ART initiation for people living with HIV regardless of CD4^+^ T‐cell count [25], mortality estimates were generated for people diagnosed with HIV since 2015. Estimates were generated for men, women and people living with HIV attributed to injection drug use. We applied benchmarks for men to IPR estimates for men living with HIV attributed to male‐to‐male sex. The IPR for each population were compared with the Australian benchmarks, as well as the UNAIDS benchmark of 0.03.

Ethical approval for this study was obtained from the University of New South Wales Human Research Ethics Committee (HC210911). The study data were deidentified prior to use in our study and consent was not sought from those notified with HIV.

## 3 RESULTS

Over the study period, for people diagnosed with HIV between 2015 and 2020, the life expectancy (D) following diagnosis was 45.7 years for women, 45.4 years for men and 45.5 years overall (Table 1). For men, women and overall, the median age at diagnosis was 35 years. Therefore, the Australian IPR epidemic equilibrium benchmark (1/D) was 0.022 for women, men and overall. For people living with HIV attributed to injection drug use, the median age at diagnosis was 39 years and the life expectancy was 37.0 years meaning that the IPR benchmark among this population was 0.028.

**Figure 1:**
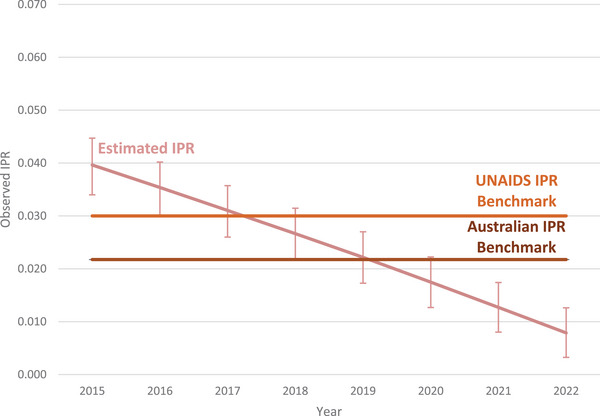
Incidence prevalence ratio among people living with HIV in Australia, 2015−2022. Abbreviation: IPR, incidence prevalence ratio.

Across the study period, the IPR estimates declined below the Australian IPR equilibrium benchmark for all reported populations. Overall, the estimate for the IPR fell by 80%, from 0.040 (range: 0.034−0.045) in 2015 to 0.008 (range: 0.003−0.013) in 2022 with the IPR falling below the UNAIDS benchmark in 2018 (IPR: 0.028, range: 0.022−0.031) and below the Australian benchmark of 0.022 in 2020 (IPR: 0.017, range: 0.013−0.022) (Figure 1).

**Figure 2:**
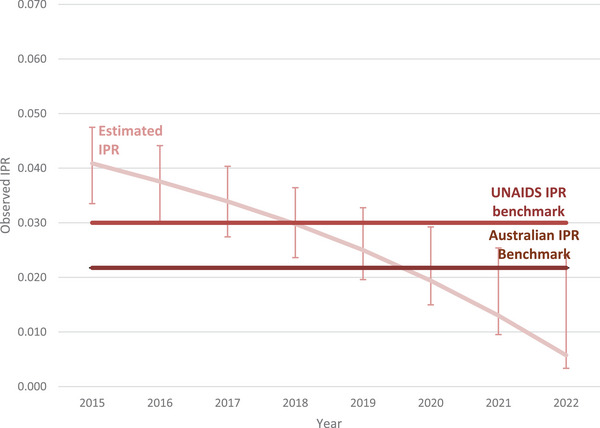
Incidence prevalence ratio among men living with HIV attributed to male‐to‐male sex in Australia, 2015−2022. Abbreviation: IPR, incidence prevalence ratio.

**Figure 3:**
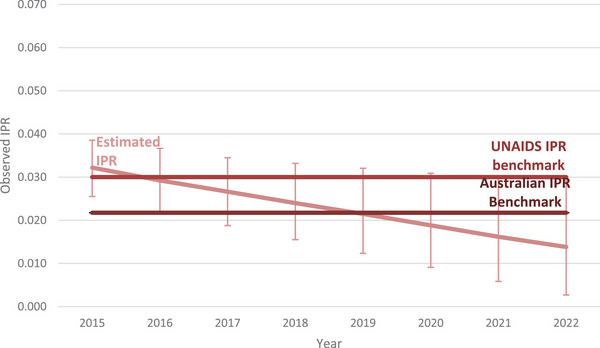
Incidence prevalence ratio among women living with HIV in Australia, 2015−2022. Abbreviation: IPR, incidence prevalence ratio.

Among people living with HIV attributed to male‐to‐male sex, the estimate for the IPR fell by 85%, from 0.041 (range: 0.034−0.047) in 2015 to 0.006 (range: 0.003−0.024) in 2022 with the IPR falling below the UNAIDS benchmark in 2019 (IPR: 0.025, range 0.020−0.033) and below the Australian benchmark in 2020 (IPR: 0.019, range: 0.015−0.029) (Figure 2). Among women living with HIV, the IPR fell by 56%, from 0.032 (range: 0.026−0.039) in 2015 to 0.014 (range: 0.003−0.029) in 2022 with the IPR falling below the UNAIDS benchmark in 2016 (IPR: 0.029, range: 0.022−0.037) and below the Australian benchmark in 2018 (IPR: 0.027, range: 0.015−0.053) (Figure 3).

**Figure 4:**
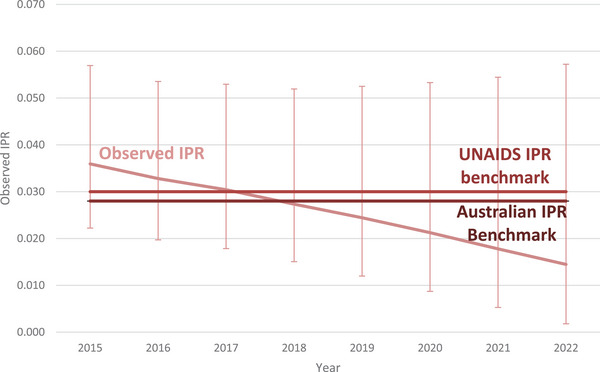
Incidence prevalence ratio among people living with HIV attributed to injection drug use in Australia, 2015−2022. Abbreviation: IPR, incidence prevalence ratio.

Among people living with HIV attributed to injection drug use, the estimate for the IPR fell by 61%, from 0.036 (range: 0.022−0.047) in 2015 to 0.014 (range: 0.002−0.057) in 2022 with the IPR falling below both the UNAIDS and Australian benchmarks in 2018 (IPR: 0.027, range: 0.015−0.052) (Figure 4). It is worth noting that for each sub‐population, small population sizes produced relatively large IPR uncertainty ranges. Consequentially, for these populations, the upper limit of the IPR estimate did not fall below the Australian benchmarks.

**Table 1:** Characteristics of HIV notifications included in life expectancy estimate calculations, 2015–2020

	Characteristic	Number of notifications	Median age at diagnosis	Estimated life expectancy^a^	IPR benchmark
Gender	Males	4763	35	44.7	0.022
	Females	556	36	45.3	0.022
	Trans and gender diverse	38	36	∼	∼
Exposure	Male‐to‐male sex	3735	34	45.4	0.022
	IDU	149	39	36.3	0.028
	Other exposures	1473	40	∼	∼
Overall		5357	35	44.7	0.022

Abbreviations: IDU, injection drug use; IPR, incidence prevalence ratio.

^a^ Expected life expectancy is the estimated number of years following HIV diagnosis.

## 4 DISCUSSION

To the best of our knowledge, this is the first study reporting IPRs against country and population‐specific IPR benchmarks. All populations reported in the study had lower IPR epidemic equilibrium benchmarks than the benchmark used in UNAIDS reporting. Despite the Australian benchmarks being lower and harder to attain than the UNAIDS benchmark, the IPR for all populations dropped below these benchmarks by the end of the study period, meaning that Australia's HIV epidemic is on the decline. This achievement may be partly credited to the widespread availability of low‐cost HIV treatment in Australia, meaning that an estimated 88% of people living with HIV (95% of those diagnosed) were receiving ART at the end of 2022 [21]. With free HIV testing combined with the introduction of ART free of charge for people ineligible for Medicare from 2023, the proportion of people living with HIV receiving ART will likely increase which may cause a further decline in the IPR [26]. The declining IPR reported here is similar to UNAIDS IPR reporting for other countries in the Asia‐Pacific region, including Cambodia, Nepal, New Zealand and Thailand [27]. However, the estimates presented here should be considered alongside indicators describing social and structural barriers to reducing the impact of HIV, including stigma, and equitable access to testing and ART [14].

The greatest decline in the IPR occurred among men living with HIV attributed to male‐to‐male sex. In 2022, this group had the lowest IPR among the reported populations, well below the Australian IPR benchmark. Because the Australian HIV epidemic is concentrated among gay and bisexual men, this population is the primary focus of Australian HIV prevention strategies. Australian gay and bisexual men have been early adopters of many HIV prevention strategies including Test and Treat and the rollout of HIV pre‐exposure prophylaxis (PrEP) [28, 29] leading to considerable declines in HIV transmission [21]. The rate of IPR decline increased over the study period which may have been associated with increasing PrEP and ART coverage during these years [21, 30]. Despite a high life expectancy for people living with HIV and the resulting low IPR benchmark compared to the UNAIDS benchmark, the declines in HIV transmission mean that the HIV epidemic among gay and bisexual men is declining.

The decline in the IPR among women was not as great as among people living with HIV attributed to male‐to‐male sex. However, among women, the IPR was the lowest of the reported populations at the start of the study period and dropped below the Australian benchmark a year earlier than among men living with HIV attributed to male‐to‐male sex. Lower HIV prevalence among heterosexuals compared with gay and bisexual men lowers the risk of HIV transmission leading to a lower HIV incidence among women. This likely contributed to a lower IPR among women across most of the study period. Increasing testing coverage and prevention measures focused on women at risk of acquiring HIV may further reduce HIV incidence among women, as well increasing life expectancy for women living with HIV, leading to decreases in the scale of the HIV epidemic among women.

The shorter life expectancy among people living with HIV attributed to injection drug use compared with other people living with HIV has been described in research analysing cohort data [13, 19]. Higher mortality among people who inject drugs is likely related to an increased risk of death from HIV/AIDS as well as from co‐morbidities including hepatitis C [19]. Similarly, our study reported a shorter life expectancy for people living with HIV attributed to injection drug use, compared with men, women and overall. This shorter life expectancy meant that people living with HIV attributed to injection drug use was the only population in the study to have a unique IPR benchmark. Despite the higher benchmark, the IPR for people living with HIV attributed to injection drug use dropped below the IPR threshold in 2019.

It should be acknowledged that for each sub‐population, the upper error range for the IPR never dropped below the IPR benchmark. This was partly due to the increasing width of the error range over the study period as the incidence estimates declined for all populations. This effect was particularly evident for women living with HIV and people living with HIV attributed to injection drug use for whom the level of HIV transmission in Australia has remained low since the start of the epidemic [21]. Although the upper error limits did not drop below the Australian benchmark, the clear declines in IPRs over the study period suggest that HIV epidemics among these populations are likely decreasing.

A strength of the method presented here are IPR benchmarks specific to key populations of people living with HIV in Australia. This aspect of the methodology addresses a concern previously raised around the use of the IPR as a metric to track HIV epidemics across regions and sub‐populations with stark differences in life expectancies [11]. This method can easily be adapted for use in other contexts where life expectancy estimates for people living with HIV are available. Another strength relates to the gap presented in Australian HIV surveillance reporting relating to HIV trends among gay and bisexual men as well as people with a history of injection drug use. Dynamic and robust population denominator estimates are unavailable for these populations which are required to generate HIV notification rates, one of the main indicators Australia uses to track the HIV epidemic [21]. In the absence of such denominators and associated notification rates, IPRs presented with population‐specific benchmarks may be used to track the changing burden of the HIV epidemic.

Some limitations of the study should be acknowledged. First, data were not available for people living with HIV stratified by place of birth or by country of acquisition. People who acquired prior to migrating to Australia comprise an increasing proportion of people diagnosed with HIV [21]. Due to variations in age at migration and its effect on age of HIV acquisition and life expectancy, there was uncertainty around the effects of migration on life expectancy estimates. Therefore, separate benchmarks were not generated for migrants to Australia living with HIV. Even so, despite the increasing estimated number of people living with HIV who acquired HIV outside Australia, the IPRs presented here offer evidence that the Australian HIV epidemic is diminishing. Similarly, data were not available for trans and gender diverse people. Future research may establish valid methods to estimate life expectancy for people living with HIV including migrants and trans and gender diverse people.

Another potential limitation with the application of this method is that changes in the age of HIV acquisition can impact the overall number of years people are living with HIV and, therefore, influence the IPR benchmark. However, in Australia, the median age of diagnosis has remained stable since at least 2013 indicating a relatively stable age of HIV acquisition [21]. Still, this trend may vary by region and could influence its adaptation for use elsewhere. Also, between‐year variations exist in the population‐level time to HIV diagnosis, and people often acquire HIV some years prior to diagnosis [21, 31]. Because age at HIV diagnosis was used as a proxy for age of HIV acquisition, the Australian IPR benchmark estimates may have been at risk of bias. If improvements in testing coverage are achieved and the average time to HIV diagnosis reduces, Australia‐specific IPR benchmark estimates will reduce, making them harder to attain. However, this would also result in reduced time to ART initiation, a reduced HIV incidence, and, therefore, a reduced IPR. A previously described method of estimating country‐specific IPR benchmarks factored in time to diagnosis. However, benchmarks for within‐country sub‐populations were not reported [30]. Future research may describe IPRs against country and sub‐population‐specific benchmarks by factoring in time to diagnosis.

Lastly, it should be noted that both the ECDC HIV Modelling Tool and methods for generating life expectancy estimates are dependent on case‐based HIV surveillance systems capable of capturing a high proportion of HIV diagnoses [16, 23]. This may mean our methods are best applied in settings where such case‐based surveillance systems have been implemented including Europe, Canada and the United States [33, 34, 35]. Our methods strengthen the argument for robust case‐based HIV surveillance systems, in alignment with UNAIDS recommendations [36].

## 5 CONCLUSIONS

HIV transmission in Australia has likely dropped below the level required to sustain the HIV epidemic among gay and bisexual men, women and people with a history of injection drug use. Led by communities adversely impacted by the HIV epidemic, Australia must persist in its efforts to minimize HIV transmission while reducing the stigma and healthcare burdens associated with living with HIV. By applying this method in other regions with robust life expectancy estimates for people living with HIV, the changing scale of HIV epidemics may be better described for populations lacking robust population denominators.

## COMPETING INTERESTS

RTG has received funding for his research from WHO and UNAIDS. RTG has provided non‐funded project advice to Gilead Sciences and ViiV Healthcare and has received speaker fees from Gilead Sciences.

## AUTHORS’ CONTRIBUTIONS

Conceptualization: JMK and SM. Data curation: HM and RTG. Methodology: JMK, HM, RTG and SM. Supervision: SM and JC. Visualization: JMK. Writing—original draft: JMK. All authors have read and approved the manuscript.

## FUNDING

The Kirby Institute receives funding from the Australian Government Department of Health and Aged Care, 4‐E0AZC3O.

## Data Availability

Australian State and Territory legislation prohibit the sharing of HIV notifications data used in this study. All cleaned publicly available data, reproducible code and analysis documentation for calculating the IPRs are available online: https://github.com/The‐Kirby‐Institute/Cascade_calculations.
